# Abnormal dot plots on current automated blood cell analyzer helped to yeast detection

**DOI:** 10.1002/ccr3.2716

**Published:** 2020-02-22

**Authors:** Delphine Gérard, Jean‐Francois Lesesve

**Affiliations:** ^1^ Service d'Hématologie Biologique University Hospital at Nancy Vandoeuvre France

**Keywords:** blood cell counter, Candidosis, Sysmex XN9000

## Abstract

Compromised data are usually flagged by instruments. This is the first report of yeast detection using the new launched Sysmex XN analyzer.

## Graphical abstract

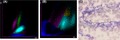

## 1 CASE REPORT

**Figure 1:**
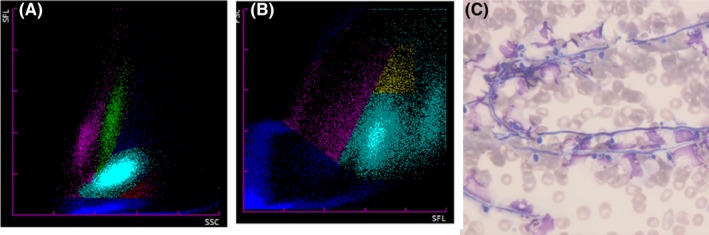
Patient 1: Abnormal lymphocytes separation (1A) on the WDF channel; dots misclassified as nucleated red blood cells (1B); and yeast observation (May‐Grünwald‐Giemsa stain, ×400, 1C)

**Figure 2:**
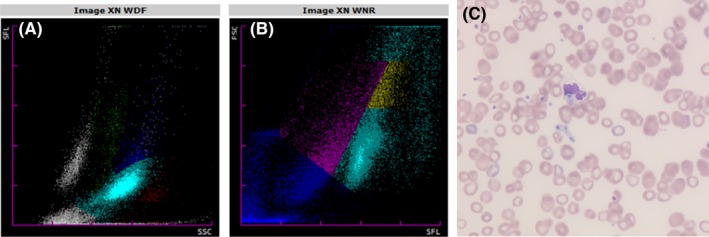
Patient 2: Nonvalidation of the lymphocyte area (2A) on the WDF channel; dots misclassified as nucleated red blood cells (2B); and yeast observation (May‐Grünwald‐Giemsa stain, ×400, 2C)

Two unsuspected cases of fungemia in a 4‐month‐old boy suffering from enterocolitis and a 47‐year‐old woman undergoing a hernia surgery are reported, with a close presentation on the blood cell analyzer Sysmex XN‐9000. White blood cell (WBC) differential (WDF) and numeration (WNR) channel histograms showed an abnormal cell population occurrence and separation: abnormal lymphocytes separation on the WDF channel (Figure 1, pink dots too close to green dots—monocytes, 1A), and notification of a nonvalidation of the lymphocyte area (Figure 2, gray dots, 2A). Abnormal clouds were detected on the left of the WNR scattergram around the debris area, highlighted in blue, and partially misclassified as nucleated red blood cells (NRBC) (Figures 1 and 2, blue dots, 1B‐2B). The data were consequently flagged for a microscopic review, leading to yeast observation for both cases (Figures 1 and 2, May‐Grünwald‐Giemsa stain, ×400, 1C‐2C) and even budding yeasts in one (Figure 2, 2C). *Candida albicans* were identified from blood culture.

The Sysmex XN uses an original technology for WBC count and differential, with separate WNR and WBC channels both using flow cytometry with semiconductor laser. The WNR channel is used for WBC, NRBC, and basophil counts, whereas the WDF channel is used for counts of neutrophils, lymphocytes, monocytes, eosinophils, and immature granulocytes. Though the analyzer is not designed to pick up fungi, the smaller and less granular shape of the yeasts as compared to WBCs justify their localization on the scattergrams, bringing up the hypothesis of possible systemic yeast infection.^1^

## AUTHORS' CONTRIBUTION

DG: collected the data, performed the analysis, and wrote the paper. J‐FL: performed the analysis and wrote the paper.
